# Non-consensus GLI binding sites in Hedgehog target gene regulation

**DOI:** 10.1186/1471-2199-11-2

**Published:** 2010-01-13

**Authors:** Martina Winklmayr, Carmen Schmid, Sandra Laner-Plamberger, Alexandra Kaser, Fritz Aberger, Thomas Eichberger, Anna-Maria Frischauf

**Affiliations:** 1 Department of Molecular Biology, University of Salzburg, Hellbrunnerstrasse 34, 5020 Salzburg, Austria

## Abstract

**Background:**

The GLI transcription factors, mediators of the hedgehog signal bind with high affinity to the consensus sequence GACCACCCA. The affinity of variant single substitutions in GLI binding sites has been measured systematically, but the affinities of the variant binding sites appears low compared to the frequency of occurrence of variant sites in known GLI target gene promoters.

**Results:**

We quantified transcriptional activation by GLI using *PTCH1 *promoter based luciferase reporters containing all single substitutions of the GLI consensus binding site. As expected variants with very low affinity did not activate the reporter. Many lower affinity binding sequences are, however, functional in the presence of moderate GLI concentration. Using two natural non-consensus GLI site promoters we showed that substitution of the variant sequences by consensus leads to comparable activity.

**Conclusions:**

Variant GLI binding sites with relatively low affinity can within natural promoters lead to strong transcriptional activation. This may facilitate the identification of additional direct GLI target genes.

## Background

Sequence specific binding of transcription factors in response to diverse cellular input signals is a major determinant in the regulation of transcription. Binding sequences for many factors have been identified by experiment and/or by a wealth of prediction methods (reviewed in [1]). Consensus binding sites were classically determined by SELEX experiments and verified by EMSA while more recently affinity measurements by methods better suited to moderate to large scale experimentation like microarray binding experiments have been used [2]. Experimentally determined affinities or frequencies for each base at every position of a binding site can be represented as position weight matrices or sequence logos, which can be used for prediction of new binding sites [3,4]. It is well known that not all sequences, which a transcription factor strongly binds to *in vitro *will also be bound in an *in vivo *context [5]. Global chromatin immunoprecipitation can identify the sequences bound by a transcription factor within the cellular context but does not indicate whether the binding site is functional, i.e. whether the presence of a given TF at this site affects expression of the target gene. For this, additional information usually derived from microarray data, sequencing or promoter studies is required [6,7].

Relative binding affinity is a good indicator of transcriptional activation or repression in an artificial system as shown for example by Kang et al for the Zif268 DNA binding domain joined to repressor or activator domains [8]. A detailed description of the quantitative relationship between affinity and activation potential in the cell is difficult since *in vivo *activation depends on the presence of co-factors, additional transcription factors and the epigenetic state of the chromatin. On the other hand, a single high affinity binding site in combination with a minimal promoter frequently does not produce strong target gene activation and reporter constructs therefore usually contain several repeats of consensus binding sites to enhance reporter activity. In the analysis of specific promoters attention is usually first focussed on consensus sites though the functionality of variant sites for many transcription factors has been shown *in vivo *and in reporter gene assays. The effect of variation in a single site on activation and specificity has extensively been investigated in *E coli*[9]. Within specific mammalian promoters the influence of variant sites on transcriptional activation has not been explored systematically.

The three GLI transcription factors, mediators of the hedgehog signal, comprise a DNA binding domain of five zinc fingers, which are very highly homologous in the three GLIs. Two of the five fingers are responsible for all but one of the protein-DNA base contacts [10]. The GLIs can function as activators and/or repressors and regulate target genes in a highly context specific way. The consensus binding sequence GACCACCCA was first determined by Kinzler et al [11] and many direct GLI target genes have been identified. Hallikas et al [12] determined the affinities of all single base substitutions in the GLI consensus binding sequence using a fusion of luciferase with the GLI-DNA binding domain in an *in vitro *assay. These data together with information on species conservation were used in the novel EEL prediction program to identify GLI regulated genes within the mouse and human genome. These predictions were successful in identifying new target genes though some known target genes were not represented in the original version. This emphasizes the need to characterise in more detail the relationship between affinity and functionality of GLI binding sites in functional assays.

We therefore set out to investigate the activity of all single site variants of the consensus GLI binding site in a luciferase assay. Frequently GLI transcriptional activity is measured in an artificial construct containing multiple copies of the consensus site. Here we use a construct based on the *PTCH1 *promoter, which is functional in many different cell types and should approximate a "normal" control of gene expression. Using relatively low GLI concentration to enhance specificity we found that a rather large number of variant GLI binding sites was able to activate transcription within the *PTCH1 *promoter. We then proceeded to turn variant binding sites into consensus within two unrelated natural promoters containing essential non-consensus GLI binding sites and found that activity was not significantly enhanced.

## Results and Discussion

### A PTCH1 reporter system to measure the functionality of variant GLI binding sites

**Figure 1:**
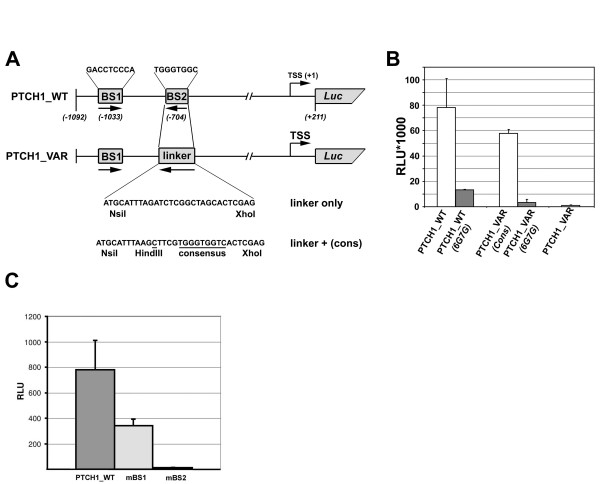
**PTCH1 reporter constructs**. (A) For PTCH1_WT a 1.3 kb fragment (-1022 to +211 relative to the main TSS) was cloned upstream of the firefly luciferase gene of the pGL3b vector. GLI binding sites BS1 and BS2 are represented by boxes, arrowheads indicate the orientation of the binding site. For PTCH1_VAR, binding site BS2 was replaced by a 29 bp linker. All variant GLI binding sites were inserted between the NsiI and XhoI restriction sites of the linker (consensus sequence is shown). (B) PTCH1_WT and PTCH1_VAR luciferase reporters with the GLI consensus binding site were co-transfected with GLI2act expression vector into HaCaT cells. Variants 6G7G (G in position 6 and 7 of consensus) were used as inactive controls. PTCH1_WT and PTCH1_VAR(Cons) show comparable activation, no activation was observed for 6G7G and the empty PTCH1_VAR construct. (C) BS2 is absolutely required for activation of the PTCH1 wild type promoter PTCH1_WT. PTCH1_WT or mutagenized variants (mBS1, mBS2) were cotransfected with GLI2act into HaCaT cells. Mutagenesis of BS2 (GACCACCCA) to 6G7G completely abolishes activation of the PTCH1_WT reporter construct (mBS2), while substitution of BS1 (GACCTCCCA) with 6G7G only reduces the luciferase signal (mBS1).

The hedgehog receptor PTCH1 is a well characterised direct GLI target gene and its elevated expression is indicative of Hh pathway activation. *PTCH1 *expression is driven from several alternative transcription start sites [13]. The *PTCH1 *promoter region upstream of exon 1B (Figure 1A) has been shown to contain a GLI consensus site (BS2, -704) [14] essential for activation by GLI. We localised a second GLI binding site (BS1, GACCTCCCA) with a single substitution compared to consensus upstream of BS2 at -1033. The presence of BS1 only is not sufficient for promoter activation by GLI in a luciferase assay, but it enhances transcriptional activation in the presence of BS2 (Figure 1C). We chose to use the essential BS2 site in the *PTCH1 *promoter to investigate the influence of all 27 possible single base substitutions in the consensus sequence on transcriptional activation. To facilitate the exchange of consensus by the variant binding site we replaced the consensus site with a linker sequence permitting the test sequence to be quickly inserted into the *PTCH1 *luciferase reporter construct (PTCH1_VAR) (Figure 1A). Together with the variant sequence, a HindIII site was inserted to allow fast identification of plasmids containing the variant sequence. The base C in position 14 relative to the start of the consensus sequence has previously been shown to positively affect GLI binding affinity [12,15] and is included in the construct as part of the *HindIII *site.

We then tested the functionality of the luciferase reporter system by comparing the ability of GLI2act to activate the reporter constructs containing the linker with the consensus sequence (PTCH1_VAR_(cons)) to the unmodified *PTCH1 *promoter luciferase reporter construct (PTCH1_WT) (Figure 1B). All results presented here were obtained with GLI2act, which is a strong activator. When GLI1 was used comparable results were obtained though activity was lower (data not shown, CS unpublished). As shown in Figure 1B both wild type (PTCH1_WT) and modified *PTCH1 *promoter construct (PTCH1_VAR(Cons)) were strongly induced in response to GLI2act with only slightly lower activation for the modified PTCH1 promoter (Figure 1B). As expected, the inactive variant 6G7G (GACCAGGCA) (Figure 1B) in PTCH1_WT as well as in PTCH1_VAR resulted in strongly reduced reporter activity. No activation was observed with PTCH1_VAR, with no inserted sequence. Thus, the modified *PTCH1 *reporter system is functional and can be used to systematically measure the effect of variation in GLI binding sequence on GLI target gene activation.

### The effect of GLI binding site variants on PTCH1 promoter activation

**Figure 2:**
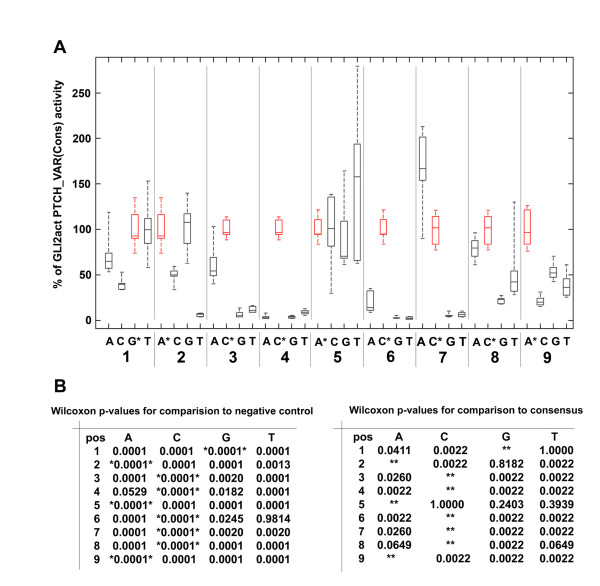
**Luciferase reporter activation by single site variants**. (A) HaCaT cells were co-transfected in triplicate with GLI2act expression constructs and the PTCH1_VAR luciferase reporter containing the consensus or a single site variant or the 6G7G (negative control). LacZ expression plasmid was added for normalization to all transfections. RLU for each transfection were first normalized with respect to lacZ expression and then with respect to the mean of RLU of three consensus samples from the same 24 well plate. The boxplot represents the results of two independent experiments normalized separately and then pooled to result in 6 measurements per position and base. Boxplots indicate the range (whiskers), estimated lower and upper quartile (box) and median (line) of the normalized values, which are expressed as percent of consensus activity. The consensus sequence in each position is coloured red and the corresponding base marked with an asterisk. A statistical analysis using an exact Kruskal-Wallis test rejects the null-hypothesis of equal population medians at significance levels p < 0.01% (that is, highly significant) for all positions with the exception of position 5, for which p = 46%. (B) Wilcoxon rank sum tests were done comparing the values of the base in each position to the negative control 6G7G (left panel) and to the consensus site (right panel). On the left, the consensus is highlighted by asterisks, on the right, the consensus positions have been left empty. p-values below 0.01 (1%) indicate clear rejections of the null-hypothesis of the medians of the distributions being equal. There are only 4 non significant values on the left, among them no single consensus. The right panel supports the boxplots in (A). Note, that due to the small sample size, only few numerical values are possible in all the (exact) Wilcoxon tests.

**Figure 3:**
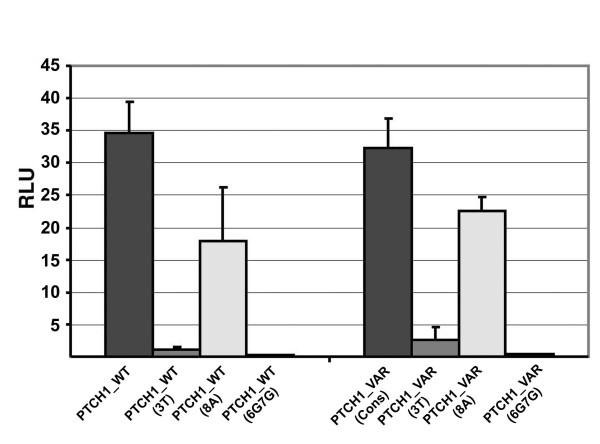
**Validation of the transcriptional activation obtained with the PTCH1_VAR construct containing a selection of different binding site variants with a modified PTCH1_WT construct in a luciferase assay**. GLI binding site variations (3T, 8A, 6G7G) were introduced into the PTCH_WT construct by site directed mutagenesis. HaCaT cells were co-transfected with the indicated reporter constructs and a GLI2act expression construct together with LacZ for normalization. No significant differences were observed between the mutated PTCH_WT construct and the PTCH_VAR construct demonstrating the relevance of the PTCH_VAR test system. 6G7G was used as inactive negative control.

To determine GLI activity for all single site variants of the 9 bp consensus binding sequence we co-transfected each PTCH1_VAR luciferase reporter together with GLI2act into HaCaT cells (Figure 2A). As a negative control we used PTCH1_VAR(6G7G) (Figure 1B). To exploit the dynamic range of the reporter system, all assays were performed under optimal transcriptional activation conditions using moderate GLI2act levels. The boxplot (Figure 2A) shows the range of activities measured at each position, statistical significance compared to negative control and to consensus is shown in Figure 2B. At first view it is striking that many sequence variants result in reporter activation similar to the consensus GLI binding site. Especially in position 5 there is no significant difference in the transcriptional activities between consensus and any non-consensus bases (Figure 2A, B). In contrast, any substitution in position 4 or 6 leads to loss of activity, consistent with affinity measurements showing complete loss of GLI binding if these critical positions are altered (CS unpublished). There are several positions where the identity of the substituted base shows a pronounced effect on transcriptional activation: in position 7 (C in consensus), G and T do not lead to reporter gene transcription while A reproducibly equals or even appears to exceed the level of activation by consensus. A number of variants results in activities intermediate between consensus and background. Taking into account the variability inherent in biological replicates it is not possible to attach significance to relatively small differences in activity. To exclude the possibility that the linker sequence, which surrounds the binding site differentially affects the activation of the various reporter constructs, we also tested a small number of binding site variants directly within the unmodified *PTCH1 *promoter construct by introducing site-specific mutations (Figure 3). No major discrepancies were observed, suggesting in summary that many variant GLI binding sites are functional and can substitute for the consensus. We then compared the transcriptional activation (Figure 2A) to the affinity profile described by Hallikas et al [12] and found that a large number of substitutions, which have quite low affinity significantly activate the luciferase reporter. This may be due to the fact that the nonlinear normalization applied to the raw data very strongly emphasizes the consensus site [16]. Conventional competitive EMSA measurements on selected binding sequences with linear normalization showed several single substitutions with Kd values within a factor of 10 of the consensus (CS, unpublished), which are compatible with the results of the luciferase reporter activity found. This is also consistent with the existence of many single and several double substitutions in the GLI consensus sequence of promoters with known GLI dependent function *in vivo *(Table 1), which failed to be retrieved in genome-wide *in silico *searches for GLI target genes [12] e.g. *BCL2*, *IL1R2*, *FST*, *TGM3 *(Table 1).

**Table 1:** Functional non-consensus GLI binding sites in Hh target gene promoters


**Gene/Promoter**	**Binding site variant**	**Number of substitutions**	**Reference**

*Gli1*	9G	1	[18]
*TGM3*	7A	1	[17]
*FST*	Bs1 3A	1	[22]
	Bs3 5G	1	
*BCL2*	Bs3 2G	1	[21]
*IL2R2*	Bs1 3T	1	[23]
	Bs2 1T2G	2	
	Bs3 1C9G	1	
*JUN*	Bs3 2G5C	2	[17]
*BMP2*	1C8A	2	[24]
*HNF3β*	3A	1	[25]
*Fgf15*	5T	1	[26]
*Fgf15*	Bs1 1T2G9G	3	[27]
	Bs2 1T2G9T	3	
*CD155*	2C	1	[28]

**Figure 4:**
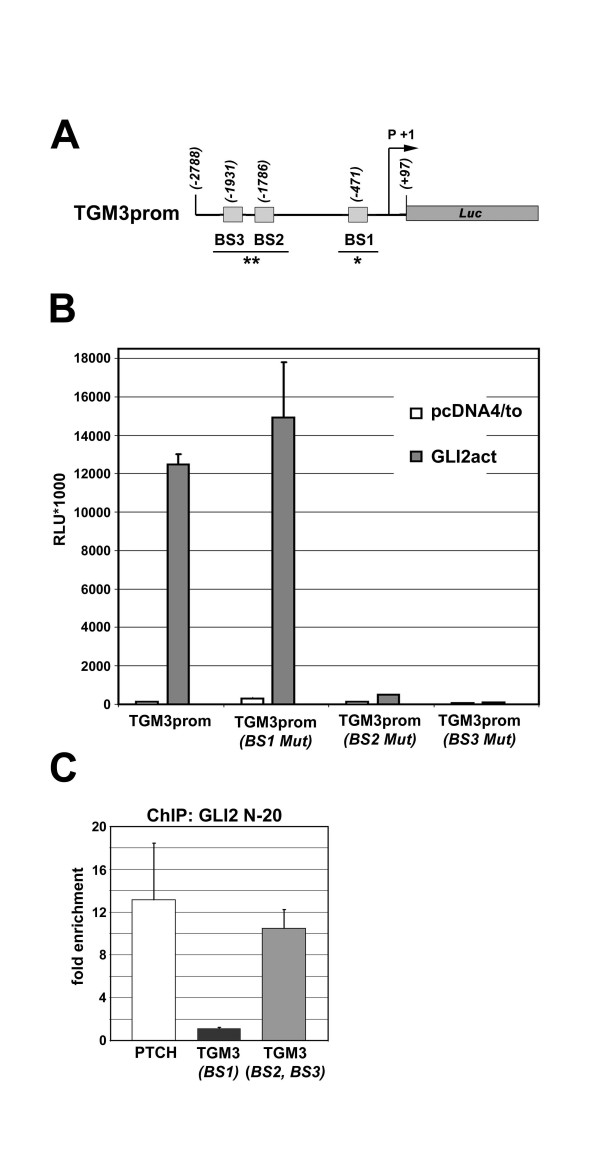
**Activation of TGM3 in response to GLI requires two low affinity GLI binding sites**. (A) The human TGM3 upstream regulatory region contains two non-consensus GLI binding sites (7A) (BS2, BS3). Lines below represent DNA amplified by qPCR in ChIP (*;, *;*;). (B) Luciferase reporter assay with the wild type TGM3 promoter fragment (TGM3prom) and constructs with mutated putative GLI binding sites (BS1 Mut, BS2 Mut, BS3 Mut). HaCaT cells were co-transfected with promoter constructs as indicated and GLI2act expression constructs or the empty expression vector pcDNA4/to. Data shown are mean values of relative light units (RLU) of three independent experiments. Mutation of either BS2 or BS3 completely abolishes reporter activation while mutation of the consensus sequence has no effect. (C) Chromatin immunoprecipitation (ChIP) demonstrates specific binding of GLI2act to a region of the TGM3 promoter containing BS2 and BS3 (*;*; in A). No amplification was observed for the region containing BS1, the GLI consensus binding site (*; in A). Chromatin isolated from GLI2actHaCaT cells was precipitated with either specific antibody (GLI2 N-20) or species matched normal IgG (nIgG). As positive control the region of the human PTCH promoter containing the functional consensus site (PTCH BS2) was used. Data shown are fold enrichment of specifically precipitated DNA (GLI2 N-20) compared to samples using species matched normal IgG for unspecific precipitation.

Though not perfectly representing the context of chromatin, luciferase reporter assays can be used to distinguish between potentially functional GLI binding sites and apparent binding sites, which do not activate reporter gene activity within their sequence context. This can be demonstrated clearly for the *TGM3 *promoter, which contains three potential GLI binding sites: one consensus sequence and two variants with a C to A substitution in position 7 (7A) (Figure 4). Mutation of the consensus sequence to nonbinding 6G7G does not affect reporter activity nor is the consensus site bound by GLI in a ChIP experiment (Figure 4B, C). In contrast, the variant sites are bound by GLI and mutation of either variant site abolishes transcriptional activation (Figure 4B, C).

### Non-consensus GLI binding sites in GLI target gene promoters

To further explore the influence of binding affinity on transcriptional activation in a natural promoter context other than *PTCH1 *we chose the *JUN *and *GLI1 *promoters, both containing functional non-consensus GLI binding sites, for further analysis

**Figure 5:**
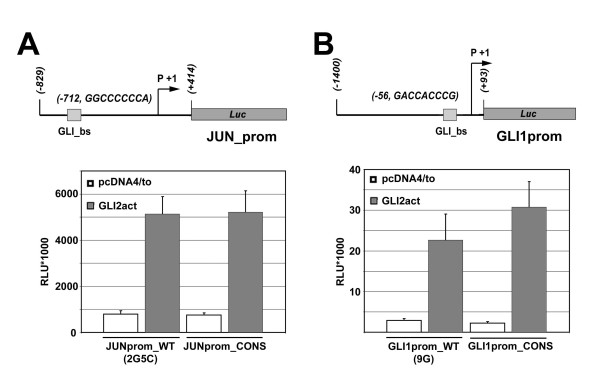
**Changing functional non-consensus sites to consensus sites in selected GLI target gene promoters does not enhance transcription**. Wild type or mutated luciferase reporter constructs were co-transfected into HaCaT cells with GLI2act expression construct or empty expression vector (pcDNA4/to) as control. (A) The human wild type JUN promoter (JUNprom_WT, upper panel) contains a functional binding site with two substitutions compared to the GLI consensus sequence (2G5C) (upper panel). The GLI consensus sequence, when introduced into this site (JUNprom_CONS), showed comparable reporter gene activation to the variant 2G5C. (B) The human GLI1 promoter contains a functional binding site with the variant nucleotide G at position 9 (GLIprom_WT, upper panel). Introduction of the consensus sequence at this position led to slightly higher activation in response to GLI2act than the wild type sequence.

The human *JUN *(JUNpromWT2G5C) [17] and human proximal *GLI1 *promoter (GLI1prom WT9G) [18] both contain only one functional GLI binding site thus eliminating possible interactions between nearby GLI binding sites (Figure 5A, B). Either binding site variant has been shown to be essential for activation by GLI and both have significantly lower affinity than the consensus site (9G coefficient according to Hallikas et al binding profile 0.004 for GLI1 (0.982 for consensus) and GLI3 (0.937 for consensus), 0.000 for GLI2 (0.982 for consensus) (see Table S1 in [12]), double substitutions as found in the *JUN *promoter were not tested under identical conditions, [12,17]. To compare the activity of the variants to the consensus, we applied site directed mutagenesis to change the wild type variant sites to the consensus sequence (GLI1promCons, JUNpromCons) (Figure 5). The luciferase reporter constructs (GLI1promCons, JUNpromCons) were then tested for the response to GLI2act in HaCaT cells and luciferase activity was compared to the respective wild type promoter constructs (GLI1promWT9G, JUNpromWT2G5C). We detected no significant difference between GLI consensus and non consensus wild type sequences in the context of either promoter. These results indicate that relatively low binding site affinity does not prevent activation by GLI in a luciferase assay at optimal GLI concentration.

Recent observations show that lower affinity binding sites for transcription factors can be identified by global ChIP [7] and occur quite frequently. Large scale affinity measurements as described in [2] showed that a large selection of transcription factors recognises many variations of the primary motifs and that even secondary motifs exist, which may possibly affect changes in transcriptional specificity. A visible influence of low affinity sites on gene expression in yeast has been described pointing to their potential relevance for modulating gene expression [19]. Vokes et al [15] identified a number of GLI promoters/enhancers, which behave in a tissue specific way and are influenced by nuclear GLI concentration. In a more global study, groups of sites with high and lower affinities to REST repressor were shown to cluster into groups responsible for activation of target genes expressed commonly, specifically or uniquely in different cell lines [20]. These data imply an important role for lower affinity sites in the context dependent control of transcription and point to the need for more detailed investigation of their function.

## Conclusion

The results presented here specifically focus on the activation potential of binding sites of the GLI transcription factors, the mediators of the hedgehog signal. We measured activation in a standardised luciferase assay in the context of the *PTCH1 *promoter testing all single site mutations of the GLI consensus binding sequence. A rather large number of substitutions was shown to be active, which is consistent with the existence of many known GLI target gene promoters containing variant sites with lower binding affinity. Taking into account the contribution of a larger subset of binding sites with significant affinity the results presented in this study are likely to be helpful in the prediction and experimental validation of more direct GLI target genes.

## Methods

### Cloning

Numbering of base positions was according to [14] for the *PTCH1 *promoter, to [17] for the *JUN *promoter and to [18] for the *GLI1 *promoter. The GLI consensus site orientation used is 5'GACCACCCA3' [11]. The wild type *PTCH1 *promoter (-1022 to +211) was amplified from BAC #RP11/43505 (obtained from Children's Hospital Oakland Research Institute (CHORI)) and cloned into the *NheI *and *BglII *sites of pGL3 basic vector (Promega, Madison, USA). For the PTCH1_VAR construct GLI binding site BS2 (-704) was replaced with a 29 bp linker sequence containing the restriction sites *NsiI *and *XhoI*. Oligonucleotides representing all variant GLI binding sites and including a *HindIII *restriction site for quick screening of positive clones were inserted into the pGL3_PTCH1_linker construct. (Figure 1A). To mutate GLI binding sites in wild type promoters we used QuickChange site-directed mutagenesis kit (Stratagene, La Jolla, USA) according to the manufacturer's protocol and verified changes by sequencing. For primers and oligos see Table 2.

**Table 2:** Sequences of oligonucleotides used for cloning, site directed mutagenesis and ChIP


**Usage**	**Primer**	**Sequence (5'-3')**

**PTCH1_WT cloning**		
	Prom_PTCH1_fw	GCAGGTCGACACACTGGCGCACTATCCAG
	Prom_PTCH1_rv	ACGAGGATCCGCTCCGGTTGACAGACCA
**PTCH1_WT mut**		
	PTCH1_mBS1_fw	GCCG**GACCT*GG*CA**GTATTTGCTGC
	PTCH1_mBS1_rv	CAAATAC**TG*CC*AGGTC**CGGCTCGC
	PTCH1_mBS2_fw	GGTTGCCTACC**TG*CC*TGGTC**TCTCT
	PTCH1_mBS2_rv	AAGTAGAGA**GACCA*GG*CA**GGTAGGC
	PTCH1_BS2-3T_fw	CACACACTGGGTTGCCTACC**TGGGTG*A*TC**TCTCTACTTTGGTGAGC
	PTCH1_BS2-3T_rv	GCTCACCAAAGTAGAGA**GA*T*CACCCA**GGTAGGCAACCCAGTGTGTG
	PTCH1_BS2-8A_fw	CACACACTGGGTTGCCTACC**T*T*GGTGGTC**TCTCTACTTTGGTGAGC
	PTCH1_BS2-8A_rv	GCTCACCAAAGTAGAGA**GACCACC*A*A**GGTAGGCAACCCAGTGTGTG
**PTCH1_VAR cloning**		
	pGL3_PTCH1_vs_fw1	AATAGGCTGTCCCCAGTGC
	pGL3_PTCH1_vs_rv1	TAGAATGCATGGTAGGCAACCCAGTG
	pGL3_PTCH1_vs_fw2	ATTACTCGAGTCTCTACTTTGGTGAGCTG
	pGL3_PTCH1_vs_rv2	GTCTTCCATGGTGGCTTTACC
	Linker_fw	TTTGAATTCCGGACCACCCAAC
	Linker_rv	TCGAGTTGGGTGGTCCGGAATTCAAATGCA
Inserted oligos*;		
	Cons_fw	TTTAAGCTTCG**TGGGTGGTC**AC
	Cons_rv	TCGAGT**GACCACCCA**CGAAGCTTAAATGCA
**Gli1_Prom mut**		
	GLI1_mBS_Cons_fw	GTTTGCGCTTCTCG**T*G*GGTGGTC**CGGGCTTGCGGCCCGGCGG
	GLI1_mBS_Cons_fw	CCGCCGGGCCGCAAGCCCG**GACCACC*C*A**CGAGAAGCGCAAAC
**JUN_Prom mut**		
	JUN_mBS3_Cons_fw	CTTCGGAGTGTTCTCAACG**TGGG*T*GG*T*C**GACTCTCGGGAGACCGC
	JUN_mBS3_Cons_rv	GCGGTCTCCCGAGAGTC**G*A*CC*A*CCCA**CGTTGAGAACACTCCGAAG
**PTCH BS2 ChIP**		
	ChIP_PTC_BS2_fwd	GAGGATGCACACACTGGGTTGCCTA
	ChIP_PTC_BS2_rev	GGGCTGTCAGATGGCTTGGGTTTCT
**TGM3 ChIP**		
	ChIP_TGM3_BS1_fw	AGAGGGGTGGGAGTGATTATC
	ChIP_TGM3_BS1_rv	AAGAAACGTCTCAGCAGAACC
	ChIP_TGM3_B_fw	AGGTCATTGAAAGGAGTGCCCG
	ChIP_TGM3_B_rv	ATGACTAAGAAAAGAGCAGAGGG

*Oligo for insertion of GLI consensus sequence (bold) into NsiI and XhoI restriction sites of the PTCH_Var promoter construct. Oligos containing all 27 GLI binding site single substitutions were designed in the same way

### Cell culture, transfection and luciferase reporter assays

HaCaT cells and GLI2actHaCaT [21] were cultured in Dulbecco's modified Eagle medium (high glucose, PAA, Pasching, Austria) with 10% fetal calf serum (PAA, Pasching, Austria) supplemented with streptomycin/penicillin (Pen/Strep100x stock solution, PAA, Pasching, Austria) at 37°C, 5% CO_2_. Cells were grown to 80% confluence in 24-well plates and transfected in triplicate with the pGL3 luciferase reporter plasmids, the GLI2act expression (80 ng/transaction sample) construct [21] or pcDNA4/TO as negative control using Superfect Transfection reagent (Quiagen Inc., Valencia, CA). LacZ expression plasmid (400 ng/transfection sample) was used for normalization of transfection efficiency. Cells were harvested 48 h after transfection, and luciferase activity measured with a LucyII luminometer (Anthos Labtec, Cambridge, UK) using Luciferase Assay Substrate (Promega, Madison, USA).

### Chromatin immunoprecipitation

ChIP from GLI2actHaCaT was done as described in [22]. Antibodies used were: polyclonal goat-anti-GLI2 (GLI2-N20) (Santa Cruz Biotechnology) for specific precipitation and species matched normal IgGs (Santa Cruz Biotechnology) for unspecific control. PCR primer sequences are listed in Table 2.

## Abbreviations

EMSA: Electrophoretic Mobility Shift Assay; TSS: Transcription Start Site; SELEX: Systematic Evolution of Ligands by Exponential Enrichment; TF: Transcription Factor; ChIP: Chromatin immunoprecipitation; Hh: Hedgehog; RLU: Relative Light Unit.

## Authors' contributions

MW and CS participated in design of the experiments and reporter constructs, carried out and evaluated luciferase assays except experiments related to TGM3, which were done by SLP, AK carried out ChIP, FA contributed to the design of the project and the evaluation of the results, TE participated in the writing of the manuscript and the evaluation of the results, AMF contributed to the design of the project, the evaluation of the results and the writing of the manuscript. All authors read and approved the final manuscript.
